# Amphibians and plant-protection products: what research and action is needed?

**DOI:** 10.1186/s12302-016-0085-6

**Published:** 2016-05-17

**Authors:** Annette Aldrich, Marion Junghans, Caroline Aeberli, Carsten A. Brühl, Franz Streissl, Benedikt R. Schmidt

**Affiliations:** 1 Agroscope, Schloss, 8820 Wädenswil, Switzerland; 2 Swiss Centre for Applied Ecotoxicology Eawag-EPFL (Ecotox Centre), Überlandstrasse 133, 8600 Dübendorf, Switzerland; 3 EWP AG, 8307 Effretikon, Switzerland; 4 Institute for Environmental Sciences, University Koblenz-Landau, Fortstraße 7, 76829 Landau, Germany; 5 European Food Safety Authority (EFSA), Pesticide Unit, Via Carlo Magno 1A, 43100 Parma, Italy; 6 KARCH, Passage Maximilien-de-Meuron 6, 2000 Neuchâtel, Switzerland; 7 Department for Evolutionary Biology and Environmental Studies, University of Zurich, Winterthurerstrasse 190, 8057 Zurich, Switzerland

**Keywords:** Amphibians, Pesticides, Ecological risk assessment, Toxicity, Exposure, Workshop

## Abstract

**Background:**

The majority of Swiss amphibians are threatened. There is a range of factors which have been discussed as possible causes for their decline, including plant protection products (PPPs).

**Results:**

The influence of PPPs on amphibian populations has not yet been studied to any great extent, neither for active ingredients nor for the wetting agents, breakdown products or tank mixtures. A further topic of discussion was how to better protect amphibians by reducing their exposure to PPPs in agricultural fields.

**Conclusion:**

Experts at a workshop concluded that further research is needed.

## Background

Plant protection products (PPPs) are biologically active substances, which means that their application in the field can have side effects on non-target organisms. In 2013, the European Union^1^ has explicitly called for amphibian toxicity data to be considered when authorising the use of PPPs. To date however, neither the EU nor Switzerland has produced any concrete suggestions or guidelines for the regulatory risk assessment of PPPs to amphibians.

The International Union for Conservation of Nature (IUCN) considers worldwide “habitat loss and degradation as the greatest threat by far to amphibians at present. The number of species impacted this way is almost four times greater than the next most common threat, pollution” [1]. PPPs fall within the category of pollution and could be important contributors, given that in Europe, the presence of some amphibian species and pesticides in fields overlap regularly [2], and there are studies detecting PPP residues in amphibians from agriculturally influenced areas [3, 4]. Unlike other contributory factors for the observed amphibian decline, PPPs undergo an authorisation process and are used deliberately, so that regulatory intervention for the protection of non-target organisms is possible.

### Expert workshop for knowledge exchange

In order to increase our understanding of the potential impacts of PPPs on amphibians (individuals and populations) and to discuss the protection of this imperiled (and protected) group of organisms, an expert workshop was held on 17 June 2015 in Dübendorf, Switzerland, by Agroscope, the Swiss Amphibian and Reptile Conservation Program (karch) and the Swiss Centre for Applied Ecotoxicology Eawag-EPFL (Ecotox Centre). The aim of this workshop was to establish a network of experts on this topic and to promote knowledge exchange between scientists, regulators, practitioners and stakeholders. The current state of knowledge was analysed by experts and unresolved issues as well as ideas for projects were gathered proactively. Given that the practitioners were from Switzerland, the situation in Swiss farmland was chosen as an example to facilitate discussions on concrete field situations.

**Fig. 1:**
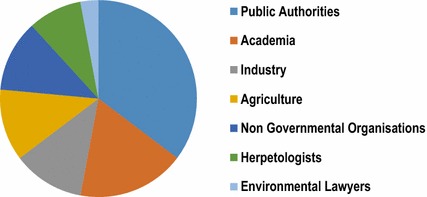
Composition of the participants in terms of their affiliation

The participants included experts and stakeholders from all involved sectors, namely academia, public authorities, agriculture, industry and environmental associations (Fig. 1). To our knowledge, this is the first workshop on this topic and within such a framework to be held in Europe.

### Current status of knowledge

As an introduction to the subject matter, lectures were given on six topics, which are outlined in brief below:

- *Natural history and endangerment of amphibians (Benedikt Schmidt, karch)* Amphibians are the most endangered class of vertebrates [5]. There are numerous reasons for their decline. The most important ones are the loss of habitat (quantity and quality), emerging diseases and the overexploitation of populations [5, 6]. In addition, various threats may interact: e.g. PPP with predators (e.g. [7, 8]) or with diseases (e.g. [9]). In the case of habitat loss, the absence of temporary ponds is a particular problem in Switzerland [10].
  PPP could also contribute to the decline, because amphibians use different habitats due to their complex life cycle and annual cycle. This means that they may come into contact with PPPs in food, water, land and air. Natterjack toads, for example, have been observed on arable land, e.g. in cereal fields, where during the day they sit on the ground or bury themselves [11]. PPPs can also be detected in amphibian breeding sites, although little data are currently available on this topic. The analysis of all Swiss Red Lists shows that aquatic species face a greater risk of extinction compared to terrestrial species [12].
  Although variability in the sensitivity of different species to PPPs has not been significantly researched to date, there seem to be certain phylogenetic patterns [13].
  The effect on the population is not necessarily predictable by observing the effects on the individual. Thus, it is often not possible to scale up from mortality of individuals to population-level effects [14]. Population models however, suggest that the survival of the postmetamorphic juveniles is of crucial importance for population dynamics [15]. Populations fluctuate greatly in size from year to year, so long-term observations are essential in order to permit an accurate statement on the impact of PPPs on amphibians [16]. Furthermore, the factors influencing population dynamics (e.g. population density or the vulnerability of various life stages) are currently not sufficiently understood to explain changes in populations.
- *Effect of pesticides on the terrestrial life phase (Carsten Brühl, University of Koblenz*-*Landau* Several amphibian species like the fire-bellied toad (*Bombina bombina)* or the common spadefoot (*Pelobates fuscus)* inhabit the agricultural landscape during their summer activity period, resulting in possible terrestrial exposure [2]. Owing to the biology of the species, it is to be expected that dermal exposure is the major route of exposure [17, 18]. The skin of amphibians has a unique structure and function that is not comparable to that of mammals. Since it is considerably more permeable, amphibians are more vulnerable to PPPs than other terrestrial vertebrates. The susceptibility of terrestrial amphibians has not yet been described to any great extent, although some studies have shown that PPPs at environmentally relevant concentrations can be toxic for terrestrial life stages of amphibians [19]. Postmetamorphic juvenile European common frogs were directly sprayed with different PPPs in a laboratory study. The mortality of the animals in the case of all seven PPP formulations investigated (4 fungicides, 2 herbicides and 1 insecticide) was high, reaching 100 % at field application rates and 40 % at 10 % field application rate, despite the fact that products and application quantities authorised in Germany and Switzerland have been tested [19]. The authors have noted that in addition to the active substance, co-formulants can also decisively influence the toxicity of PPPs to amphibians.
- *Ecotoxicological risk assessment for the authorisation of PPP (Annette Aldrich, Agroscope)* In Switzerland, the Ordinance on Plant Protection Products (PSMV) forms the legal basis for authorisation, and the same data requirements and assessment criteria apply as in other European countries, with the effects of the individual PPP on birds, mammals, arthropods, non-target plants, soil macro- and microorganisms, fish, aquatic invertebrates and aquatic plants being investigated. Surrogate species of the various groups are tested as representatives of all organisms. However, it is the aim of the PSMV that PPP have no unacceptable effects on the environment as a whole, and on non-target organisms in particular. Until now, direct tests with amphibians have not been required. It was assumed that risks to tadpoles could be assessed by considering toxicity data from surrogate aquatic organisms such as, e.g. fish, and that effects on terrestrial amphibians could be assessed by considering toxicity data from surrogate terrestrial vertebrates such as birds or mammals. To enable assessment of the declared aim of the PSMV, the protection goal must be defined, and relevant information regarding exposure and toxicity must be available. Ultimately, risk is assessed by comparing toxicity and predicted exposure. Both parameters are based on results from standardised studies and models, so that extrapolating to the actual condition in the environment is fraught with uncertainty. The more the situation to be assessed differs from the studied situation, and the smaller the number of studies, the greater the uncertainty. This means that although it seems that the sensitivity of tadpoles to PPPs is comparable to that of fish [20, 21], an accurate statement cannot be made on the risk of PPPs for aquatic amphibians, since no specific information is yet available on exposure, nor is the variability in sensitivity known. At present, we do not yet know how the sensitivity of various species, populations and life stages varies, and which species could serve as representative organisms. It is likewise still unclear how amphibians take up PPPs, and how exposure can be calculated with models. Finally, it should be considered that amphibians represent a strongly imperiled group of organisms for which it seems mandatory to minimise any additional stress in order to avoid population collapse. Because of the unanswered questions on risk assessment for amphibians in the authorisation process, one should think about how specific measures can be used to reduce exposure to PPPs, thereby increasing the level of protection. The migration corridors in the amphibian spawning areas of national importance are mentioned here as an example of places in which the use of PPPs is to be monitored, particularly in the spring. In principle, the regulatory authority can mandate risk mitigation measures (obligations) through which a potential risk can be reduced.
- *Coincidence of pesticide application and presence of amphibians (Carsten Brühl)* Amphibians can come into direct contact with PPPs on agricultural land in two ways: firstly, by inhabiting cultivated fields, and, secondly, by crossing the areas in question on the way to spawning or after spawning and metamorphosis. Because of their annual cycle (migrating to their breeding sites and back, foraging in their summer habitat), amphibians can be found on agricultural fields over the entire six-month summer activity period and are thus potentially directly exposed to PPPs, although interception by plants may seasonally reduce exposure. The percentage of individuals in a population active on arable land during PPP application varied between 0.8 and 74.6 %, depending on species and year of study [2]. Particularly high percentages were affected in the case of PPP use in winter cereals and winter oilseed rape. In many cases, species were present during several applications of insecticides, herbicides or fungicides. The likelihood of exposure is lower for species migrating to their breeding sites early in the year than for those migrating to the ponds later in the year [2].
- *EFSA activities concerning the preparation of a risk assessment of PPP for amphibians (Franz Streissl, EFSA)* The European Food Safety Authority (EFSA) is developing guidance documents for environmental risk assessment of PPP in Europe. The evaluation of the effects on amphibians is an important topic which will be part of a future Guidance Document. A scientific opinion summarising the state of the science on the risk assessment for amphibians and reptiles will provide the scientific basis. EFSA organises rounds of public consultation in order to have the feedback of the different stakeholders. As a preparation, a report was drafted on the sensitivity, occurrence, habitat use and exposure of amphibian species in agricultural environments [18]. The authors concluded that absorption through the skin is likely to be the main route of exposure. Toxicity data for terrestrial species or life stages are scarce, and therefore, it is difficult to estimate their sensitivity to PPPs. Based on available data, the sensitivity of tadpoles seems to be comparable to fish [20, 21]. Aquatic life stages occurring in permanent waters are therefore covered by the existing first tier risk assessment for fish [22]. EFSA initiated a study to collect data on population effects and toxicity data for terrestrial life stages, and compared these with toxicity endpoints observed in studies with standard test organisms. The aim is to use toxicity endpoints of standard test organisms as a surrogate for the risk assessment of amphibians to avoid additional toxicity tests with amphibians. A further aim is to be able to extrapolate effects observed in the laboratory to population-level effects in the field.
- *The precautionary principle (Caroline Aeberli)* The precautionary principle is a way of dealing with scientific uncertainty, based on available scientific knowledge, the degree of uncertainty and societal values (e.g. legal or political assessments). In Switzerland, the precautionary principle is anchored in the Federal Constitution (BV) and the Environmental Protection Act (USG). The aim of the USG is to protect people, animals and plants, as well as their biotic communities and habitats, from harmful effects or nuisances. Pursuant to art. 1 paragraph 2 of the USG, *“In keeping with the precautionary principle, […], impacts which could become harmful or create a nuisance are to be limited at an early stage”*. The precautionary principle also applies in further decrees, *inter alia* in the Water Protection Act (GSchG) and the Chemicals Act (ChemG), or at ordinance level, for example, in the Plant Protection Product Ordinance (PSMV). The precautionary principle is chiefly underpinned by the concept of avoiding or limiting incalculable risks. It creates a safety margin (in Swiss legal terms) taking the uncertainties vis-à-vis the longer-term effects of environmental pollution into account.^2^ The precautionary principle can be applied in the form of measures even when there is not yet any concrete danger, and it is intended to be effective where scientific uncertainty still exists. This principle is meant to provide preventive protection against risks as well as environmental protection focused on the long term (“prevention instead of cure”). According to Tschannen,^3^*“a plausible probability, based on empirical values, that the impacts could become harmful or a nuisance in the foreseeable future…”* is sufficient. Owing to its legal nature, however, the precautionary principle is to be classified as strongly programmatic, which means that there will always be a debate about the ‘what’ and ‘how’. This even applies when it has already undergone a process of concretisation at, e.g. ordinance level. Thus, in article 1 para. 4 of the PSMV, reference is made to the precautionary principle as the basis for the provisions of the PSMV, and the legislation for the placing on the market of PPPs (art. 14 PSMV) represents a fleshing-out of the precautionary principle. In these cases, the precautionary principle is already considered, and the taking of further-reaching measures whilst invoking the precautionary principle is difficult in legal terms [23]. This does not, however, mean that the precautionary principle cannot be applied in a specific case or cannot be used in a further-reaching manner within the scope of revisions of the concrete definitions. Thus, articles 148a and 165a of the Law on Agriculture stipulate in what instances precautionary measures can be taken, what form these may take and how long they are to apply. In principle, the plausibility of an unacceptable side effect must exist, and the likelihood of its occurrence must be rated as substantial or its consequences must be far reaching. The precautionary principle applies essentially in the same way in the EU.

## Discussion

After the lectures, participants were divided into two groups in order to discuss the following questions:

- How high do the experts estimate the risk potential of PPPs for amphibians to be in the field?
- What research questions should be addressed to support the development of a risk assessment scheme for amphibians?
- What measures can be implemented on a voluntary basis or on the basis of the precautionary principle to reduce pressure on amphibian populations in agricultural areas?

The participants had time before the group discussions to formulate their own ideas on the issues, so that everyone had the opportunity to contribute. Their ideas and questions formed the starting point for the group discussions. The aim was to collect the full range of opinions, not to reach a consensus. After each question, participants could rate, and hence prioritise, the individual ideas. Below, the breadth of the suggestions is summarised, with the spontaneously favoured ideas by the participants shown in the box.

### Risk potential

Exposure and effects are the factors that determine the risk posed by PPPs to amphibians. As regards to the effects, it was largely uncontested that PPPs can have a toxic effect on amphibians. What was disputed, however, was to what extent amphibians are exposed in the field and how well the existing experimental studies reflect this exposure. Whilst some participants noted a temporal and spatial coincidence of amphibians and PPPs, others observed that there was still a lack of studies taking particular account of the behaviour of amphibians (e.g. burying behaviour). It was pointed out that multiple factors besides PPP such as habitat loss, diseases and predators could contribute to the amphibian decline. The relative contribution of PPPs for amphibian population declines is currently unclear and it was not the aim of the workshop to discuss all factors for amphibian decline. However, field observations suggest that there are important factors besides the loss of habitat [6]. According to the statements of participants from the amphibian protection sector, in some areas even apparently suitable ponds are not inhabited. In the opinion of these participants, the possible influence of PPPs should be more closely investigated in these cases. The unique life cycle of amphibians, with both an aquatic and terrestrial phase, as well as the measured PPP residues in waters [24] and the permeable amphibian skin put them at potential risk from PPPs. PPPs can also represent an additional stress for amphibians already at risk, and any additional stress can be problematic. These are all observations leading to the conclusion that PPPs pose a risk, especially in agricultural landscapes, although the extent of this risk compared to that posed by other factors is unclear. On the other hand, it was mentioned by some participants that they know of successful conservation projects in which no particular emphasis was placed on the reduction of exposure to PPP. It was pointed out that studies have often been carried out with active ingredients that are no longer approved and that studies with current formulated products would be helpful. Another issue brought up was that toxicity studies using direct overspray at field application rates might not represent a realistic exposure scenario, because interception by the crop canopy will likely reduce the exposure of the amphibians.

After the discussion, workshop participants (for background, see Fig. 1) were invited to give their spontaneous assessment of the topic. Here, they were given the choice between three responses: (i) “There is a problem”. (ii) “It is unclear whether or not there is a problem, but it is an interesting topic”. (iii) “There is not a problem”. The query was made on a flipchart, and each participant had one sticker to place by their selected response. According to the results, 58 % of the participants thought that there was a problem, and hence a need for action. A further 33 % rated the topic ‘effects of plant-protection products on amphibians’ as interesting. Only 8 % of the participants saw no problem, and hence no need for action.

**Table:** 

What, in your opinion, is indicative of the presence or absence of a significant threat to amphibians from PPPs?	
Presence of threat:	
Spatial and temporal coincidence of PPP application with the presence of amphibians	
Exposure in water and on land owing to their special life cycle	
Laboratory studies in which effects are observed at full and reduced application rate	
Population declines in nature	
Absence of threat:	
Uncertainty in terms of actual exposure	
Relative impact of other factors	
Relevance to population trend is unclear	
Exposure reduced by crop canopy	

### Research issues

In agreement with EFSA, it was deemed necessary to define the protection goal for amphibians in greater detail, and in particular to evaluate the relevance of effects on populations. Therefore, it can be helpful to develop population models that allow us to assess long-term effects at population level on the basis of effects on individuals. To this end, it was suggested that observations in the field are increased. Which species and developmental stages are exposed in which crop to which PPP at what point in time, and to what extent must be determined. Coincidence studies, in which the application of PPP is correlated with the presence and behaviour of the species, are helpful for this, but direct, indirect and large-scale exposure in the field should also be observed in order to develop exposure models. There are still many unanswered questions regarding the toxicity of PPPs for amphibians, including co-formulants, mixtures, multiple stressors and interactive effects. Is it possible to predict the effects according to the mode of action of the PPP? Effects on terrestrial life stages have still not been studied enough. The question of which species is most suitable for testing is also still unresolved. Many studies are carried out with the African clawed frog, but its representativeness for native species has been questioned by the participants. The variability between species and populations should be studied so that uncertainty and applicability may be estimated. For an efficient risk assessment for amphibians, it was proposed to define entry criteria to identify quickly problematic active substances and co-formulants on the basis of certain substance properties. Standard inhalation toxicity studies with birds and mammals might be helpful to identify substances which could be acutely toxic to amphibians via dermal exposure, since the percutaneous absorption rates of substances through the amphibian skin is deemed to be very high. Ultimately, risk mitigation measures should be available so that the risk both inside and outside the agricultural field can be reduced. Part of this consists e.g. in research on cultivation methods without the use of PPP. The effect of buffer strips to reduce the input into ponds should be investigated, as should the design of habitats, e.g. existing biodiversity-promoting areas (a type of agricultural set-aside), so that they too benefit the amphibians. How other factors, e.g. tillage or mechanical weed control, affect the amphibians, and the extent to which these factors contribute to the decline of the amphibians compared to the impact of PPPs was also discussed. A weighing of the various factors should be conducted. Interactions between factors make it difficult to deal with them separately. Here, developments in research on multiple stressors should be pursued. A further overarching issue was addressed: What approach or approaches to protect amphibians are the most efficient in terms of costs (for the farmer) and effect (on the amphibians)?

**Table:** 

What research issues should be addressed?	
How can realistic exposure of amphibians to PPPs be assessed?	
How great is the risk to which species, at what developmental stage, in which crop and from which PPP?	
Which effects on individuals and at what life stages are most relevant for population trends?	
Which population models can be used to estimate the effects observed in the laboratory with individuals, at the population level as a whole?	
What effect do co-formulants have on toxicity?	
How high is the variability in sensitivity between the various amphibian species and populations?	
Can entrance criteria be developed in order to identify especially problematic substances for ecotoxicological risk assessment in the authorisation of PPP?	
What risk-management options are there both inside and outside of the field?	
How relevant are other factors for the decline in the amphibian population, compared to PPPs?	

### Risk mitigation measures

In the discussion on measures which could be taken, the topics of authorisation, reduction/optimisation of PPP use, advisory and awareness-raising activities and habitat improvement were at the forefront. For authorisation, one important topic was the need to adapt risk assessment schemes in order to identify PPPs with unacceptable affects and prevent their authorisation. Furthermore, it was hoped that comparative assessments would allow differentiation between problematic and less problematic active substances or formulations and rank them according to their risk potential. The aim would be for the industry to reduce or withdraw problematic applications or PPPs from the market, or to develop recommendations on how and when the use of the PPP in question could be reduced or avoided in a professional or private context. The ‘advisory and awareness-raising activities’ topic took up the latter subject, determining that farmers should be informed of the dangers of PPP use for amphibians particularly where amphibians migrate from land habitats to ponds. It was stressed that advice is also necessary in the context of PPP use in domestic gardens. Advice and increasing awareness could also lead to a reduction in and optimisation of the use of PPP. Approaches such as alternative plant protection, abstention from the use of certain PPP, or the use of PPP strictly in accordance with Integrated Production (IP) rules would be conceivable here. Where mechanical alternatives to PPP use are recommended, the possible influence of these alternatives on amphibians should also be considered. One mitigation measure could be based on the prediction of amphibian migration so that PPP are not used when amphibians are present in large numbers on farmland. A variety of measures under the heading ‘habitat promotion’ were discussed—for example, untreated corridors could be set up on farmland where amphibians are present or to which they migrate. Direct payments to farmers could be a possibility to promote the implementation of amphibian friendly management measures including amphibian-specific ecological compensation areas or biodiversity-promoting areas, or even in IP agriculture. Particular attention should also be paid to the amphibian breeding sites of national importance, as well as the migration corridors (e.g. a ban on application, even if only temporary). The question of costs and benefit was also of importance in the subject area of ‘measures’.

**Table:** 

What measures can be implemented voluntarily or on a precautionary basis to reduce pressure on the amphibian population?	
Avoidance of PPP application during migration in affected areas with the help of prediction models	
Recommended avoidance of specific products in vulnerable areas or critical time periods	
Advice and awareness raising for farmers	
Advice and awareness raising for private users in their home gardens	
Compensation measures (e.g. amphibian-specific ecological compensation areas)	
Promotion of amphibians via direct payments and/or existing label schemes, e.g. IP-Suisse	
Reduction of PPP use through alternative plant protection strategies where possible and sensible	

## Conclusions and outlook

The workshop showed that there is a need for additional knowledge on the subject of amphibians and PPP, and that this topic should be pursued further. Much information was exchanged and many ideas were gathered and contacts established. The broad-based cooperation between stakeholders from agriculture, industry, environmental associations, public authorities and herpetologists has proven useful, and must be developed further. Workshop participants were made aware of the topic of amphibians and PPP, and this awareness must now be carried on into the various areas represented at the workshop. Seemingly crucial is the question of whether further research is necessary in the first instance, or whether preventive measures should be taken in order to improve the situation for the amphibians. Speaking for the latter is the fact that amphibian populations are at risk; for the former, a higher level of knowledge will allow measures to be applied in a more strategic and effective fashion, as the relative contribution of the different PPP to the risk is not yet quantified. The protection of amphibians should be considered particularly when elaborating new concepts, e.g. as part of the National Action Plan for risk reduction and sustainable use of PPP, as well as in existing concepts concerning agricultural policy, such as the Direct Payment Ordinance (specific incentives to remunerate farmers for services of public and common interest). At the end of the workshop, it was proposed that a pilot project be launched to gather more information on the exposure of amphibians to PPP in the field. Such a project requires good collaboration, the foundation of which was laid during the workshop.

## Footnotes

1 Commission Regulation (EU) No 283/2013 of 1 March 2013 setting out the data requirements for active substances, in accordance with Regulation (EC) No 1107/2009 of the European Parliament and of the Council concerning the placing of plant protection products on the market, and Commission Regulation (EU) No 284/2013 of 1 March 2013 setting out the data requirements for active substances, in accordance with Regulation (EC) No 1107/2009 of the European Parliament and of the Council concerning the placing of plant protection products on the market.

2 see decisions BGE 124 II 219 and BGE 131 II 431 of the Swiss Federal Court.

3 Tschannen, USG commentary, Art. 1 marginal no. 33; Vereinigung für Umweltrecht/Keller Helen (Publ.), Kommentar zum Umweltschutzgesetz, 1st and 2nd editions, Zurich 1998 ff.
